# Talking about depression: a qualitative study of barriers to managing depression in people with long term conditions in primary care

**DOI:** 10.1186/1471-2296-12-10

**Published:** 2011-03-22

**Authors:** Peter A Coventry, Rebecca Hays, Chris Dickens, Christine Bundy, Charlotte Garrett, Andrea Cherrington, Carolyn Chew-Graham

**Affiliations:** 1 Greater Manchester Collaboration for Leadership in Applied Health Research and Care, Health Sciences Research Group and Manchester Academic Health Sciences Centre, Williamson Building, University of Manchester, M13 9PL, UK

## Abstract

**Background:**

The risk of depression is increased in people with long term conditions (LTCs) and is associated with poorer patient outcomes for both the depressive illness and the LTC, but often remains undetected and poorly managed. The aim of this study was to identify and explore barriers to detecting and managing depression in primary care in people with two exemplar LTCs: diabetes and coronary heart disease (CHD).

**Methods:**

Qualitative in-depth interviews were conducted with 19 healthcare professionals drawn predominately from primary care, along with 7 service users and 3 carers (n = 29). One focus group was then held with a set of 6 healthcare professionals and a set of 7 service users and 1 carer (n = 14). Interviews and the focus group were digitally recorded, transcribed verbatim, and analysed independently. The two data sets were then inspected for commonalities using a constant comparative method, leading to a final thematic framework used in this paper.

**Results:**

Barriers to detecting and managing depression in people with LTCs in primary care exist: i) when practitioners in partnership with patients conceptualise depression as a common and understandable response to the losses associated with LTCs - depression in the presence of LTCs is normalised, militating against its recognition and treatment; ii) where highly performanced managed consultations under the terms of the Quality and Outcomes Framework encourage reductionist approaches to case-finding in people with CHD and diabetes, and iii) where there is uncertainty among practitioners about how to negotiate labels for depression in people with LTCs in ways that might facilitate shared understanding and future management.

**Conclusion:**

Depression was often normalised in the presence of LTCs, obviating rather than facilitating further assessment and management. Furthermore, structural constraints imposed by the QOF encouraged reductionist approaches to case-finding for depression in consultations for CHD and diabetes. Future work might focus on how interventions that draw on the principles of the chronic care model, such as collaborative care, could support primary care practitioners to better recognise and manage depression in patients with LTCs.

## Background

People with chronic physical health problems or long term conditions (LTCs) are approximately twice as likely to suffer from depression than the adult general population [1,2]. Furthermore, when present with LTCs, depression is significantly associated with greater reductions in health status compared with depression alone, or with single or multiple LTCs alone [1]. This is especially important from a therapeutic perspective because depression is linked to poorer self-care [3], noncompliance with medical treatment [4], and disengagement from lifestyle and behavioural changes known to be protective in people with LTCs [5].

Despite evidence that supports the efficacy of antidepressants and structured forms of psychotherapy, depression generally remains under-detected and under treated by non-psychiatric health professionals, including general practitioners (GPs) [6]. There is growing recognition that the identification and management of depression is similarly problematic in the presence of LTCs. In the UK, the National Institute of Health and Clinical Excellence (NICE) have published guidelines in 2009 that incorporate stepped care models to facilitate the delivery of accessible and effective treatments for depression in people with LTCs [7]. Further, since 2006 in England, the quality and outcomes framework (QOF) of the general medical services contract has incentivised GPs to screen for depression in diabetes and coronary heart disease (CHD) [8]. However, in UK general practice, rates of antidepressant prescribing remain lower for older patients and patients with LTCs (including CHD and diabetes) and recognised depression [9]. Even where GPs are aware of risk factors for psychological comorbidity, there is evidence that time constraints, a lack of confidence in diagnostic acumen, and a perception that patients resist discussing emotional problems and avoid taking antidepressants hinder the management of depression in people with LTCs [10]. Overcoming these problems remains a challenge.

The reasons why primary care practitioners apparently struggle to recognise, diagnose and then manage depression in people with LTCs remain uncertain. We undertook a qualitative study, using in-depth interviews and a focus group with stakeholders in primary care (professionals, patients and carers), of barriers to the management of depression in two exemplar LTCs: diabetes and CHD. This exploratory work forms the first phase of a programme funded through the National Institute for Health Research Collaboration for Leadership in Applied Health Research and Care (CLAHRC). We have focused in this paper mainly on the barriers and problems encountered by professionals engaged in managing depression in people with diabetes and CHD. The results will inform the development and implementation of innovative strategies within the research programme to support and facilitate primary care practitioners to effectively detect and treat depression in people with LTCs.

## Methods

### Interview participants and recruitment

We purposively recruited a diverse sample of stakeholders in order to elicit views on a wide range of barriers to the management of depression in people with LTCs [11,12]. Healthcare professionals were indentified from NHS trusts in Greater Manchester using a shortlist of relevant job roles, and invited to participate by letter. Patients with diabetes and/or CHD were recruited by distributing details of the study to healthcare professionals and volunteer organisations. Interested patients contacted the research team directly or, with consent, had their details passed on. Carers present during patient interviews were asked if they would like to participate, and interested individuals were interviewed either with the patient, after the patient interview had finished, or on a separate occasion. Before each interview the aims of the study were explained verbally and informed consent was obtained in writing.

### Data collection

This exploratory study used in-depth, semi-structured interviews. The schedules consisted of a list of key topic areas with open ended questions and additional prompts and explored the following areas: awareness and vulnerability to depression in LTCs, presentation and detection, management of depression, communication in the consultation, and training and development to improve services for depression in LTCs (see Additional files 1 and 2).

All interviews were carried out between January and August 2009 by two of the authors (RH and PC). The interviews took place at participants' homes or places of work, the researchers' place of work, and a community centre. Interviews were digitally audio-recorded and transcribed verbatim with consent. One respondent agreed to be interviewed but did not give consent for recording; detailed field notes were taken by the interviewer.

### Data analysis

Data analysis was concurrent with data collection to enable the incorporation of relevant emerging themes into subsequent interviews and the seeking of deviant cases [13,14]. A thematic approach was used, incorporating principles of constant comparison, to categorise emergent and recurring themes within and between transcripts. Themes were identified using an open-coding method. A preliminary coding frame was constructed using the interview schedules to structure the themes. This was then adapted in accordance with emerging data as linked codes were grouped to form categories. Disconfirming evidence and deviant cases were sought throughout the analysis [13].

Analysis was carried out by 3 researchers from different backgrounds (general practice, health services research and psychology) to increase trustworthiness of analysis [15]. Transcripts were analysed independently, then emerging themes were discussed until consensus was achieved. Each transcript was analysed individually and then in groups, with the healthcare professional (providers and commissioners/managers) transcripts analysed separately from the patient and carer transcripts but with comparisons made across data sets.

### Data triangulation: focus group

In order to assess the validity of the interview analysis, preliminary findings were presented to new and existing participants at a focus group, in June 2009. Individuals who had previously been interviewed and expressed an interest in continued involvement in the research were invited to participate. Additional recruitment took place using the same shortlist of job roles devised for the interviews. The healthcare professionals and patients/carers participated in separate facilitated group discussions about barriers to managing depression in people with LTCs. With consent, these discussions were audio-recorded and transcribed verbatim. Analysis of the transcripts was conducted in the same way as for the interview data. The data from the focus group served to triangulate the data collected from interviews, removing uncertainty about the interpretation of findings from the interviews.

### Ethical approval

The interview study (08/H1004/150) was given ethical approval by Salford and Trafford Local Research Ethics Committee; and the focus group study (09/H1015/20) was given ethical approval by Cumbria and Lancashire A Research Ethics Committee.

## Results

In-depth, semi-structured interviews were carried out with 19 healthcare professionals, 7 patients and 3 carers. The focus group was attended by 4 of the original interview participants and an additional 5 healthcare professionals and 5 patients (see Tables 1 and 2).

**Table 1:** Health Care Professional (HCP) Participant Demographics for Interview (I) and Focus Group (FG) Studies

HCP	Study	Profession	Age	Gender	Ethnic group
HCP01	I	GPSI in CHD	51-60	Male	Mixed race

HCP02	I	Advanced Practitioner	41-50	Female	White British

HCP03	I	GPSI in Mental Health	51-60	Male	White British

HCP04	I	GP	51-60	Male	White British

HCP05	I	Practice Nurse	41-50	Female	White British

HCP06	I	Diabetes Nurse	41-50	Female	White British

HCP07	I	CHD Nurse Clinical Lead	41-50	Female	White British

HCP08	I	GP (with ethnic minority interests)	31-40	Male	British Asian - Bangladeshi

HCP09	I	Primary Care Mental Health Worker	21-30	Female	White British

HCP10	I	Consultant in Psychological Medicine	51-60	Female	White British

HCP11	I	Equality and Diversity Advisor	51-60	Female	African-Asian

HCP12	I	GP (with ethnic minority interests)	41-50	Male	British-Asian Pakistani

HCP13	I	GPSI in Mental Health & PBC	51-60	Female	White British

HCP14	I	GPSI in Diabetes	41-50	Male	White British

HCP15	I	Consultant Diabetologist	41-50	Male	White British

HCP16	I	Clinical Psychologist	41-50	Male	White British

HCP17	I	Occupational Therapist	41-50	Female	White British

HCP18	I+FG	Associate Director for Primary Care Commissioning	41-50	Female	White British

HCP19	I	Manager of a Third Sector group	41-50	Male	British-Asian Indian

HCP20	FG	Practice Nurse	51-60	Female	White British

HCP21	FG	GPSI in Mental Health	31-40	Female	White British

HCP22	FG	Primary Care Mental Health Worker	21-30	Female	White other

HCP23	FG	Liaison Psychiatrist	31-40	Male	Indian

HCP24	FG	Locality and Project Manager	31-40	Female	White British

GP : general practitioner; GPSI: general practitioner with special interest.

**Table 2:** Service User (SU) and Carer (CA) Participant Demographics for Interview (I) and Focus Group (FG) Studies

SU/CA	Study	Patients' LTC	Age	Gender	Ethnic group
SU01	I*	Diabetes	51-60	Female	White British

SU02	I	CHD	61-70	Male	White British

SU03	I+FG	CHD	71-80	Male	White British

SU04	I	Diabetes and CHD	61-70	Female	White British

SU05	I	CHD	61-70	Male	White British

SU06	I+FG*	CHD	61-70	Male	White British

SU07	I**	Diabetes and CHD	61-70	Female	Asian Pakistani

SU08	FG**	Diabetes and CHD	51-60	Female	British Asian

SU09	FG	Diabetes and CHD	51-60	Male	British Asian - Pakistani

SU10	FG	Diabetes	61-70	Female	White British

SU11	FG	Diabetes and CHD	41-50	Male	African-Asian Indian

SU12	FG	Diabetes	51-60	Female	Black African-Caribbean-British
					

CA01	I	CHD (SU02)	61-70	Female	White British

CA02	I+FG	CHD (SU03)	61-70	Female	White British

CA03	I*	CHD (SU05)	61-70	Female	White British
					

* Interviewed alone - other interviews carried out with carer or service user present

** Discussion conducted through an interpreter

Key themes that emerged from both the interviews and focus group suggest that barriers to effective patient centred care for depression in people with LTCs are manifest: i) when professional and patient narratives about awareness of and vulnerability to depression in the presence of LTCs lead to the normalisation of distress; (ii) where there is uncertainty among professionals about moving from awareness of emotional problems to formal screening for depression in the context of QOF; and iii) where there is uncertainty about how professionals and patients negotiate labels for depression. In this paper we draw primarily on professional perspectives from the interviews, and include patient and carer data where relevant. Data from the focus group is used to supplement findings from the interviews. Illustrative data is given and identified by respondent number and interview or focus group.

### Awareness and heightened vulnerability to depression

There was widespread recognition among GPs that the consequences and symptom burden of LTCs can account for heightened vulnerability to depression among people with diabetes and CHD. GPs often conceptualised depression in LTCs in comparable terms to late-life depression - as 'inevitable' and as a 'normal' consequence of living with a LTC:

"Once you're told you've got some sort of chronic disease it upsets your equilibrium, it upsets the way you see yourself, you lose your autonomy in terms of your health. It impacts on your family. So all of those are bound to have...a psychological impact on...you as a patient." [HCP01]

"...the disability and the handicap that they [LTCs] cause, or the intrusion certainly...weigh heavily on patients' minds..." [HCP14]

The notion that depression in people with LTCs is inextricably linked to losses associated with physical decline and possibly old age was reflected in patients' accounts about their mental health:

"...sometimes it comes over me sometimes and I think well you know I was so fit, why has all this happened to me you know? And I have a bit of a cry ... it's a very hard pill to swallow when you've been so active." [SU04]

"...at my age when I went through the change your emotions do change anyway, it's all about getting older and accepting that you are getting older and...and I do think that plays a big part..." [SU01]

Compared with GPs, nurses and allied health professionals articulated their understanding about the relationship between mental health and LTCs in more equivocal terms. Whilst many construed depression as an understandable reaction to living with a LTC, some nurses acknowledged the possibility that precipitating factors for depression might also stem from social and economic disadvantage:

"...sometimes it's not because of the diabetes, sometimes it's because of life itself, we have a credit crunch on, so things are getting worse. And we work in an area here that's got high unemployment and high social problems...I don't necessarily know whether it's [depression] because of the disease, or because of their social conditions." [HCP05]

Clinicians suggested that depression in people with LTCs was insidious in onset and this might account for why these patients (and doctors) sometimes struggle to discriminate between changes in their mood and their general state of ill health:

"I think they could well not recognise the symptoms [of depression] in themselves, particularly if they've been quite slow onset...I think if you've had something major that's happened and you've gone into a deep depression you recognise there's a huge great big change, whereas if something's quite slow it's just kind of accepted as part of your everyday life. And no they don't recognise it then." [HCP05]

Clinicians also suggested that variation in patients' ability to recognise depression might be explained by the tendency for patients with diabetes and chronic heart problems, (especially people from black and minority ethnic [BME] groups), to attribute psychological symptoms to disability associated with their physical condition:

"...somebody's got heart failure, say mild heart failure, but as a result [their] breathing isn't as good. Feeling depressed, their breathing will be worse, rather than feeling it as a depression." [HCP03]

"I think sometimes they [BME patients] might attribute their symptoms [of depression] to the...illness itself, rather than to the consequences of the illness..." [HCP01]

In articulating their awareness of heightened vulnerability to depression in diabetes/CHD, clinicians co-constructed with patients a normalising narrative about depression that might obviate rather than facilitate efforts to move towards formal identification and later, treatment of depression as a separate clinical entity.

### From awareness to identification and assessment

In primary care in England, screening for depression in diabetes and CHD is incentivised by the QOF [8]. Screening for depression was described as taking place during annual review consultations, often by nurses, but the GPs who took part in this study reported various levels of engagement in the identification and management of depression in people with LTCs. Opinion amongst GP respondents was divided about the extent to which the QOF had changed working practices associated with identifying mental health problems, especially in the context of consultations for diabetes/CHD. Some GPs cautiously embraced opportunities to more routinely screen for depression using tools such as the Personal Health Questionnaire (PHQ-9) [16]^:^

"...one of the good things that's happened recently with QOF targets and parameters...is to screen for depression so its something that we are now actively doing and may not have done four or five years ago" [HCP12].

Other GPs were more equivocal in their judgements about how the QOF had changed screening and assessment practices, highlighting concerns that its introduction had, at best, formalised practices that were already largely routine, especially for those with an interest in mental health and, at worse, led to a more bureaucratic and less engaged approach to identifying depression:

"...most of them [GPs] are not actually doing anything new they are just doing it in a slightly different way now to make sure that they get the points and a lot of that is just simply recording what they were already always doing..." [HCP08]

In the context of busy and demanding annual check-ups, some practice nurses, along with GPs, suggested that screening for depression in people with diabetes/CHD might be relegated to the margins and become something to be disposed of to free up time for other QOF priorities:

"If you've then identified...depression on a PHQ-9 then go onto...diabetes...which increases your workload again because there's a lot of other things that now with depression you've got to do for QOF, so you're increasing your workload, so as well as everything else, you've not got resources and you're also increasing your own workload." [HCP20_FG]

Aside from logistical issues, efforts to formally identify depression using tools recommended under the QOF were also compromised where patients' first language is not English. This was true for multi-lingual clinicians as well as for clinicians who only spoke English:

"... they [PHQ-2/9] are very difficult to translate and they are very difficult to use in certain languages and I am not very linguistically gifted...sometimes I really struggle and sometimes I ask very basic questions but I can't really go into it..." [HCP12].

However, although clinicians highlighted the ways in which QOF-centric practices might militate against effective screening in patients with LTCs, there was equal voice given to the notion that management of diabetes/CHD in primary care afforded opportunities for frequent contact with patients not possible in outpatient clinics in secondary care:

"...even though it may only be ten minutes at a time and they feel that they get a brief time with us I still think you know, four, ten minute appointments in six weeks does give you a degree of rapport whereas three months in a hospital and you might see a junior doctor one of those times and a consultant so you don't have continuity..." [HCP14]

Continuity of care is a key principle of general practice, but thought to be especially important in managing people with long term, life-limiting illnesses [17]. In developing longitudinal relationships with patients with diabetes/CHD, clinicians suggested they could weave into their consultations more subtle and responsive methods of identifying depression that privileged judgement and intuition over formal screening:

"...if I had seen the patient a few times and got to know them and got the feeling that all the problems they are having are you know, I would bring it up, either just asking them if they felt down or how are they feeling about it all..." [HCP08]

Equally, whilst there might be pockets of resistance to formal assessments of depression, there was an understanding among GPs that screening for depression with the PHQ in diabetes/CHD could be a positive experience for patients:

".. some patients just don't like doing it, but I think most people do, especially because I think it validates...their symptoms and also reinforces the fact that we are taking an interest in them." [HCP14]

In describing strategies to identify depression in diabetes/CHD participants drew attention to tensions between work practices that favoured disposal of screening in the context of annual check-ups, and practices that blended formal assessments with patient centred approaches. How clinicians resolve these countervailing tensions can subsequently affect the next steps along a pathway towards agreeing management of depression in patients with LTCs.

### Negotiating the label - depression as a negotiated identity

Clinicians described how patients with LTCs with identified depression commonly resisted using mental health labels to describe changes in their mood. This was particularly true of patients who were thought to be somatising or who attributed their low mood to old age or illness, but also of patients who perceived depression as a sign of weakness or of 'letting themselves down':

"...it's a sign of failure I think to think you've got depression, that you're not coping, and people don't like to be looked at that way, and therefore they will say 'I'm fed up'...then they'll answer yes to both of our screening questions. And I say, well do you think, is there any chance you could be depressed? 'Oh no, no, I'm just really, really fed up'." [HCP05]

Where depression was perceived as long standing as opposed to of recent onset, clinicians described patients as equally reticent to draw on a narrative that reinforced their depression as a recognisable entity. Moreover, GPs acknowledged that there is a tendency to collude with patients in avoiding talking about depression, especially where patients have a history of disengagement with services or where patients are thought to be overwhelmed by other, long term co-existing problems:

"I've got quite a few patients [with LTCs] I can think of who've got long term mental health problems who just don't really want to address them. And it takes an effort to, well I suppose there's two issues, one is do I want to spend the time raising it? Two is, is it kind, or is it intrusive?" [HCP04]

For their part some patients acknowledged that they rarely prompted their GPs to talk about depression, preferring to label it as transient in nature and more akin to low mood:

"...if I do go to see him [the GP], it's usually about something else and I don't really think to say anything about it [the depression]...because it is not something that is happening continually...it is something that happens now and again and you just get low with it...to be honest I've not really told him [the GP] you know. [SU04].

All participants who had experience of serving immigrant and ethnic minority groups recognised that deficits in language skills, on the part of both practitioners and patients, can lead to unsatisfactory and difficult discussions about screening results and depression diagnoses:

"I think it's harder to...[talk] about mental health issues with people who struggle to communicate in English, because that's how I know how to communicate. It's difficult to know if they're understanding what you're asking, and then conversely I'm not sure I'm understanding what they're trying to tell me." [HCP01]

Clinicians suggested that depression as a label or concept might not be readily assimilated by patients from BME groups, leading to concerns that talking about mental health might invoke culturally inappropriate language:

"...I guess you have to be careful with the language you use because you...could cause offence." [HCP08]

Patients from BME groups acknowledged that unlike physical health, cultural prohibitions about mental health or psychological labels meant that conversations about depression were fraught with difficulty, often leaving them with no one to turn to:

"...it's the stigma that is attached to the psychology aspects of it. If you go and say you're diabetic, then that is accepted and it's easier to engage with...But when it comes to the issue of depression, then it's a whole different ball game. If I'm feeling depressed, who do you talk to about it?" [SU09_FG]

Where patients' first language was not English, GPs reported using interpreters, but this approach only partly resolved communication problems. Rapport and trust between doctors and patients were seen as key to facilitating discussions about depression, but clinicians noted that consultations with interpreters (even in secondary care where there are dedicated link workers) often lacked transparency and necessitated a reliance on non-verbal communication:

"I think certainly with any sort of emotional work, its very difficult, you don't know...what's actually being said...I suppose you have to use other clues more ...you are more attentive to body language and facial expression and things like that...it can be very difficult...when there is a big conversation going on between the interpreter and the person and only two words come back to you and you don't know what the rest has been said..." [HCP13]

As well as differences in language, problems talking about depression with patients from BME groups might also relate to fears among patients that interpreters (and GPs from the same ethnic minority) might breach confidentiality:

"...there is a stigma attached to saying that they [BME patients] have a mental health problem...there may also be some issues about people not appreciating how confidential...the consultation is so there might be some anxiety around disclosing information that they think might actually...be disseminated into the wider public, into communities..." [HCP08]

To overcome barriers that are partly attributable to language and cultural difference, clinicians described ways of talking about depression that moved beyond a focus on semantics to include descriptors that were meaningful and acceptable to patients:

"...the way I would approach it would be to try and find the language, I don't just mean sort of English versus Urdu, I think its more a question of trying to find a way of explaining things to people that makes sense to them..." [HCP13]

To discuss depression in ways that resonated and had salience with patients' own understandings about mental health, clinicians described techniques they used which avoided using explicitly psychological or diagnostic labels:

"...I don't want to take a patient down a psychological path if there's no need to...With some patients also, 'well it's stress' that's all they need to know, that's all they want to know...'stress' covers a whole host of sins..." [HCP03]

However, clinicians acknowledged that by talking about depression in ways that were familiar and acceptable to patients they might over simplify their message:

"You do your best, like you can minimise it a bit too much sometimes you know, by sort of, saying...'are you feeling a bit down at the moment?' it makes it sound...[like] it's not that serious. It's sometimes hard to get a balance by not wanting to scare people off by saying 'depression' and not wanting to trivialise it or minimise it..." [HCP21_FG]

For groups that were perceived as most resistant to biomedical labels for depression such as older adults or people from BME communities, GPs, especially those with mental health expertise, described using allegorical methods such as story telling or figurative language to facilitate discussions about depression:

"I mean in mental health you use quite a lot of metaphors because it's an easier way of just explaining, because there's no pictures, no scan, so I sort of use a metaphor, sort of like a kind of picture." [HCP03]

Agreeing a label was therefore seen by practitioners as a key step towards supporting patients with LTCs manage their depression:

"...you really need them to get them to buy into the fact that, first of all do they agree that they are depressed, if they agree, do they agree that it is a situation that they want to improve and do they feel that I can improve their lot and sometimes they won't accept the diagnosis or they don't accept that there is anything that needs doing." [HCP14]

Ultimately, regardless of which approach was used to discuss mental health problems, clinicians stressed that opportunities to treat depression in the presence of LTCs rested in part on patients' ability and willingness to conceptualise depression as an identifiable and manageable problem.

## Discussion

### Main findings

Practitioners and patients differed in their ability to explain and recognise depression in the presence of LTCs, but both engaged in attributional styles that normalised distress. In the context of highly managed and time-limited consultations the normalisation of depression in people with LTCs underscored tensions between template driven approaches to case-finding, and strategies that were less regimented and more patient centred. Further, in addition to logistical issues, differences in language, culture and conceptual models about depression restricted practitioners ability to negotiate and agree labels with patients with LTCs about managing the depression.

### Limitations

The samples for both the interviews and focus group included only a relatively small number of practitioners per professional role. However, given that LTCs are often managed by multi-professional teams the samples for the interviews and focus group reflected the broad range of roles that are engaged in the delivery and commissioning of primary care services for people with LTCs. Secondly, patients and their carers are under-represented in the samples for the interviews. In this study, our primary aim was to generate data about barriers to depression care in LTCs drawn from professional perspectives, but to support these findings with data from service users and carers. This is because professional perspectives are likely to be critical to informing service redesign and training interventions to improve quality of depression care in LTCs. Service user perspectives figured more prominently in the focus group and will do so in future evaluations of such interventions as part of the CLAHRC programme. Category saturation was not achieved in the data-set of patient/carer interviews. Thirdly, some participants were involved in both the interviews and the focus group and their opinions may well not be representative of professionals and users not engaged in research. Additionally, participants who had previously been interviewed could have been sensitised to the broad aims and objectives of the study in ways that may have biased their assessments and contributions during the focus group. However, conducting a focus group after in-depth interviews is in keeping with methodological triangulation whereby different techniques and perspectives are used to investigate a single problem, removing uncertainty about the interpretation of preliminary findings [18].

### Comparisons with previous research

A range of patient, practitioner, and structural and organisational factors are known to collectively or independently constrain optimal management of depression in primary care. Under-recognition might stem from patient attitudes and beliefs about depression that can shape attributional styles that relegate depression to either somatic symptoms, or as a normal response to difficult life events [19]. For their part, professionals' conceptualisations of depression as normal and justifiable, especially in older adults and those with difficult lives, underscores therapeutic nihilism in primary care [20]. Unlike in other primary care encounters where normalisation may be contested by patients and lead to a cascade of (possibly unwarranted) diagnostic and therapeutic interventions [21], we found that professionals and patients co-constructed a normalising discourse about depression that militated against its recognition in the presence of a LTC. This points up the salience of previous findings about professionals' reluctance and patients' reticence to discuss depression in the context of LTCs [10]. It also supports the notion that whilst personal continuity in primary care can be positive for patients with long term and complex conditions, it can also be the basis for dysfunctional relationships [22], in which practitioners collude with patients they are (over) familiar with to avoid talking about depression.

Whilst acknowledging that patient-practitioner relationships and attributional styles are key to the recognition of depression there is strong support for understanding how they are shaped and constrained in the context of wider structural and organisational features of primary care. In the UK NHS, logistical challenges associated with time-limited consultations, the separation of mental health services from general practice, and poor access to psychological services hinder the provision of integrated and effective treatment for depression in primary care [23]. Given these structural constraints, patients' subjectivity and opportunities for collaborative approaches to managing depression potentially recede in the face of abbreviated consultations that centre on logistics and disposal [24,25].

In the early 21^st ^century NHS, the introduction of the QOF has accentuated tensions in primary care that arise from GPs rhetorical commitment to holistic and patient centred care and a real shift towards disease oriented and biomedical models of care [26]. As we highlighted, practitioners are more equivocal than patients in their acceptance of the use of depression severity questionnaires [27], with some preferring to draw on clinical intuition and more patient centred approaches to diagnosis. But, despite practitioners' espousal of patient centredness, we found that in the context of consultations for LTCs, the QOF fostered reductionist strategies through the use of depression screening questionnaires. During annual physical check-ups, discussion of depression may be pushed to the margins, creating barriers to more patient centred approaches to recognition and management. Whilst screening tools and checklists can confer advantages in time limited consultations, fee-for-service approaches such as the QOF can create barriers to negotiated strategies for managing depression in LTCs [28]. This is echoed in work around primary care for LTCs where reductionist approaches driven by templates and fragmented division of labour marginalises the ability of practitioners to develop self-management dialogues with patients [29].

### Implications - facilitating improved depression care in LTCs

Our work draws attention to two areas that are candidates for intervention: i) optimising patient-practitioner interactions during consultations to elicit shared understanding about the presence of depression in LTCs and ii) reconfiguring the structure and organisation of primary care services to facilitate collaborative management strategies for depression in LTCs.

Educational interventions that focus on implementation of guidelines have demonstrated limited success in primary care [30]. Rather than design more skills based training centred on technical proficiency and knowledge acquisition we would support the notion that it is first important to understand how GPs (and other practitioners) conceptualise and explain depression as these formulisations are likely to shape their approach to diagnosis and management [31]. This points to developing training and educational interventions that support practitioners to draw on their expertise and knowledge in ways that allow them to articulate and craft explanations about symptoms in partnership with patients [32]. Importantly, training that facilitates a more critical and self-aware approach to explanation need not focus on language differences between patients and practitioners. Rather, the focus here should be on how to support practitioners to engage with and empower patients through shared conceptual language.

Although we did not exclusively focus on issues related to minority groups, many of the professional participants in this study expressed frustration about their inability to effectively use tools such as the PHQ to identify depression among patients drawn from immigrant and minority ethnic groups. However, when working with patients from BME communities, much depression work need not focus on translation of tools and words alone, but focus instead on negotiating an agreed path through multiple and contested perspectives on emotional distress [33]. Metaphors and figurative language may be important tools to do this [34], especially when working with BME patients [35].

To be effective however, training interventions need to be delivered alongside organisational and systemic changes to the delivery of depression care for people with LTCs. Furthermore, remodelling the organisation of depression care is more likely to be effective when underpinned and guided by mature and theoretically informed frameworks [36]. Given that multi-morbidity is common in primary care there is growing awareness that financial incentives and guidelines that lead to an emphasis on single diseases should be displaced by more generic models of care, such as the Chronic Care Model [37]. Originating in the United States, the chronic care model appeals to whole system perspectives in which health care systems are seen as the main barrier to delivering effective treatments for LTCs. Successful elements of the chronic care model include collaborative problem determination and goal setting and better self-management support. The Improving Mood Promoting Access to Collaborative Treatment programme has demonstrated that an adapted chronic care model can improve depression in older adults [38]. Whilst the TEAMcare study has shown that collaborative care that draws on the principles of the chronic care model significantly improves depression and medical outcomes in people with CHD and diabetes [39]. There is now scope for extending the chronic care model to improve depression care among people with a range of LTCs [40], which is the next step of the CLAHRC programme.

### Conclusion

Barriers to detecting and managing depression in LTCs relate to patients' and practitioners' failure to recognise depression, a propensity to normalise distress in the face of LTCs, and the way highly performance managed environments in primary care militate against shared understandings about depression. Improvements in the quality of care for depression in people with LTCs are likely to follow on from interventions and service redesign that support and facilitate practitioners to engage patients in more collaborative management strategies.

## Competing interests

The authors declare that they have no competing interests.

## Authors' contributions

PC, RH and CCG designed the study; PC and RH collected and analysed the data and drafted the paper. CCG analysed the data and contributed to writing the paper. CG and AC collected focus group data, and with CB and CD contributed to writing the paper. All authors read and approved the manuscript.

## Pre-publication history

The pre-publication history for this paper can be accessed here:

http://www.biomedcentral.com/1471-2296/12/10/prepub

## Supplementary Material

Additional file 1 **CLAHRC Interview topic guide - patients**. Topic guide used with patients and carers during in-depth interviews Click here for file

Additional file 2 **CLAHRC Interview topic guide - Professionals**. Description: Topic guide used with professionals during in-depth interviews. Click here for file
