# Supplementary material for: Talking about depression: a qualitative study of barriers to managing depression in people with long term conditions in primary care
Source: BMC Fam Pract. 2011 Mar 22;12:10. doi: 10.1186/1471-2296-12-10 (PMC3070666; doi:10.1186/1471-2296-12-10)
Supplement: Additional file 1 — CLAHRC Interview topic guide - patients. Topic guide used with patients and carers during in-depth interviews [file 1471-2296-12-10-S1.DOC]

| ***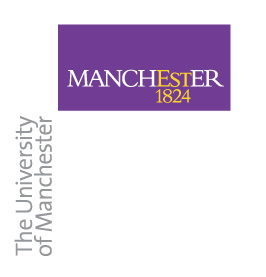*** | 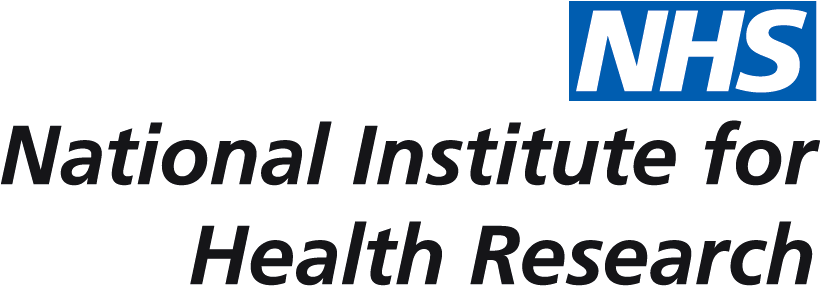  **Collaboration for Leadership in Applied Health Research and Care for Greater Manchester** |
| --- | --- |

**Barriers and facilitators to the treatment of depression in people with diabetes and CHD – informing the development of care pathways**

**CLAHRC Interview Topic Guide: Patients**

**Introduction**

- Review of Participant Information Sheet
- Timing and confidentiality

**About You**

| **You** | Can you start by telling me a bit about yourself and your background? |
| --- | --- |
| Age | How old are you – 30’s, 40’s… ? |
| Ethnicity/  Religion/  Language | What ethnic or cultural group do you belong to?  What is your religion?  What languages do you speak and read/write in? |

**Long Term Conditions**

| **Condition** | You were invited to participate in the study because you have [NAME OF CONDITION]. Can you tell me when your illness started? |
| --- | --- |
| **Management** | Could you describe the main features of the care you receive for your diabetes/CHD?  e.g. who (GP, clinic nurse/doctor)  e.g. where (GP clinic, hospital clinic, community/third sector)  e.g. when (routine/booked appointments, length of appointments) |
| What | What happens when you go to see the doctor/nurse/other? |
| Differences | Do you think your experience is the same as other people’s?  Do you think your HCPs take account of your age/culture? |
| **Accessing care** | Have you ever encountered difficulties getting help for your diabetes/CHD? If so, what difficulties did you have? |
| Solutions | Did you manage to overcome the difficulties you experienced or can you think of ways in which these problems might be overcome? |
| Differences | Do you think people from different backgrounds have different experiences of trying to get help for diabetes/CHD? |
| **Treatment preferences** | What is your opinion of the treatment you receive? |
| Effectiveness | What has worked best, what has been least successful and why do you think that was the case? |
| Preference | Ideally, what type of treatment would you like to receive? Who from, where and when? |
| Input | Do you feel that you have contributed to and had input into treatment decisions and are you happy with this level of contribution? |
| **Communication** | Do you feel like your healthcare practitioners understand you and listen to what you have to say? |
| Problems | Have you experienced any difficulties communicating with your healthcare practitioners? |
| Differences | Do you think your experience would be different if you had a different background? |

**Emotional and Psychological problems**

| **Experience** | Have you felt anxious or stressed at all during the past few months?  Have you felt low or depressed recently? |
| --- | --- |
| Cause | Do you think that your diabetes/CHD contributed to this?  What else do you think contributed to this? |
| Coping with LTC | Many people experience difficulties adapting to and living with diabetes/CHD – have you ever found it difficult to cope with?  e.g. excessive worry (leading to sleep disruption), negativity/pessimism, hopelessness, feeling overwhelmed. |
| Past experience | Prior to being diagnosed with diabetes/CHD, had you experienced any emotional or psychological problems? |
| **Management** | Since your diagnosis, have you received treatment for an emotional or psychological problem such as anxiety or depression?  What did you receive treatment for? |
| Treatment | Could you describe the main features of the care you received?  e.g. what – what type of treatment(s) did you receive?  e.g. who – who delivered the treatment?  e.g. where – where did you receive treatment?  e.g. when – when was it offered and how long did it last? |
| Past experience | Had you received treatment for an emotional or psychological problem prior to your diagnosis of diabetes/CHD?  If so, how did this differ from the more recent treatment(s) you have received? |
| Differences | Do you think your experience is the same as other people’s?  Do you think your HCPs take account of your age/culture? |
| **Accessing care** | Since your diagnosis of diabetes/CHD, have you encountered difficulties getting help for emotional or psychological problems? If so, what difficulties did you have? |
| Solutions | Did you manage to overcome the difficulties you experienced or can you think of ways in which these problems might be overcome? |
| Differences | Do you think people from different backgrounds have different experiences of trying to get help for emotional or psychological problems? |
| Past experience | Where there any differences in your ability to access help before and after your diagnosis of diabetes/CHD? |
| **Treatment preferences** | What was your opinion of the treatment(s) you received? |
| Effectiveness | What has worked best, what has been least successful and why do you think that was the case? |
| Additional  support | Have you benefited from other forms (more informal forms) of coping and support?  e.g. family/carer/support group/alternative therapy |
| Preference | Ideally, what type of treatment would you like to receive? Who from, where and when? |
| Input | Do you feel that you have contributed to and had input into treatment decisions and are you happy with this level of contribution? |
| **Communication** | Do you generally feel able to talk about emotional/psychological problems with others? |
| Who | In the past, who have you spoken to about emotional/psychological problems?  e.g. your family/friends/GP/other health care professionals/other? |
| When | Do your healthcare practitioners ask about how you are coping? If so, how do you feel being asked about this? |
| Problems | Have you experienced any difficulties communicating with your healthcare practitioners with regard to how you are coping?  In your experience would you say that healthcare practitioners focus on managing the physical symptoms of your diabetes/CHD or the way in which you cope with or adapt to it? |
| Differences | Do you think your experience would be different if you had a different background? |
