# Supplementary material for: Talking about depression: a qualitative study of barriers to managing depression in people with long term conditions in primary care
Source: BMC Fam Pract. 2011 Mar 22;12:10. doi: 10.1186/1471-2296-12-10 (PMC3070666; doi:10.1186/1471-2296-12-10)
Supplement: Additional file 2 — CLAHRC Interview topic guide - Professionals. Description: Topic guide used with professionals during in-depth interviews. [file 1471-2296-12-10-S2.DOC]

| ***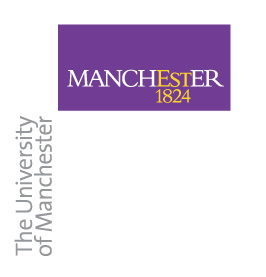*** | 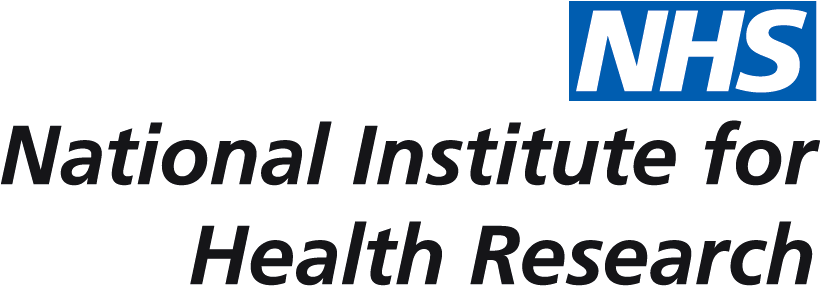  **Collaboration for Leadership in Applied Health Research and Care for Greater Manchester** |
| --- | --- |

**Barriers and facilitators to the treatment of depression in people with diabetes and CHD – informing the development of care pathways**

**CLAHRC Interview Topic Guide: Healthcare Practitioners**

**Introduction**

- Review of Participant Information Sheet
- Timing and confidentiality

**You**

| **Role and experience** | To start with, could you tell me a bit about your current role and what you do here at [NAME OF WORKPLACE]? |
| --- | --- |
| Organisation | How does your role fit within the organisation?  What other healthcare professionals are there? How many? |
| Treatment population | What types of patients/service users do you see?  e.g. chronic disease, marginalised groups, South Asians  Are there any groups that you feel you don’t see? |

**Psychological problems in LTC**

| **Prevalence** | In your experience how common are psychological/psychosocial problems in patients with chronic disease?  What about in diabetes and CHD? |
| --- | --- |
| **Type** | What types of psychological problems do you typically see in patients with chronic disease?  What about in diabetes and CHD? |
| Differences | Does the prevalence and type of psychological problem vary in relation to what chronic disease a person has? How? Why?  Does the prevalence and type of psychological problem vary in relation to any other patient characteristic? How? Why?  e.g. marginalised groups, South Asians |

**Presentation and detection**

| **Presentation** | Do you think patients with chronic disease recognise when they experience psychological symptoms?  If so, how do they seem to view their psychological symptoms? |
| --- | --- |
| Reporting | Do patients self-report psychological symptoms?  Do they do this with or without being prompted? |
| Differences | Do you think different groups perceive psychological problems differently?  e.g. marginalised groups, South Asians |
| **Screening** | Do you routinely screen for depression as part of your management of chronic diseases?  What about in diabetes and CHD?  When do you screen? How often? |
| How | How do you recognise/screen for depression?  Do you use screening questionnaires?  If so, how do you use them? |
| QOF | Has the introduction of the QOF changed the way you go about detecting and screening for depression in patients with chronic diseases? |
| Differences | Do you have different approaches for different groups?  e.g. marginalised groups, South Asians |

**Management**

| **Treatment options** | Could you talk me though your typical response to a patient who presents with depression and diabetes/CHD?  e.g. When - When would treatment be offered?  e.g. What - What are the treatment options?  e.g. How – How do you decide which treatments to offer?  e.g. Who - In what way does the patient play a role in making decisions? |
| --- | --- |
| Follow-up | What arrangements would you make for follow-up?  What might you do if someone didn’t return for follow-up? |
| Continuity of Care | When dealing with complex cases how important do you rate continuity of care and opportunities to develop longer-term relationships with patients?  Do you feel you can offer continuity of care? How? Why (not?) |
| Differences | Do you adopt different treatment approaches with different groups?  e.g. marginalised groups, South Asians |
| **Guidance** | How does what you do relate to current guidance?  e.g. NICE, stepped care |

**Communication**

| **Communication** | What factors do you think affect patient professional communication? |
| --- | --- |
| Depression | Can you think of any occasions when you have experienced problems communicating with people with depression? |
| Chronic disease | Do you think it is different/more difficult communicating with people with depression who also have a chronic disease? |
| Differences | Are there specific issues to consider during consultations with different patient groups?  e.g. marginalised groups/South Asians |
| Impact | How much do you think the ability to communicate clearly with a patient affects the quality of the treatment they receive? |

**Training and development**

| **Training** | Of the training you have had, what has been useful for the management of patients with depression and a long term condition? |
| --- | --- |
| Training gaps | Do you think that there have been gaps in your training in relation to this patient group? |
| Differences | Did your training address differences between patient groups?  e.g. marginalised groups, South Asians |
| **Development** | What would help you better manage this patient group? |
| Training | Can you think of any training or learning objectives that might help improve quality of care? |
| Other | Can you think of any tools you may find useful?  e.g. treatment pathways, guidelines |
